# CAR T-cells vs. bispecific antibodies as third- or later-line treatment for relapsed/refractory follicular lymphoma: a literature review and meta-analysis

**DOI:** 10.3389/fimmu.2025.1611984

**Published:** 2025-09-29

**Authors:** Ying He, Ling Qiu, Dan Chen, Shi-hui Ren, Ya-xin Xiong, Meng-jiao Li, Bai-tao Dou, Yan-ling Li, Ya-li Cen, Yun-ming Li, Hao Yao, Fang-yi Fan

**Affiliations:** ^1^ Department of Hematology, Chinese People’s Liberation Army The General Hospital of Western Theater Command, Chengdu, Sichuan, China; ^2^ Department of Hematology, National Clinical Research Center for Hematological Disease, Chengdu, Sichuan, China; ^3^ Department of Hematology, Sichuan Clinical Research Center for Hematological Disease, Chengdu, China; ^4^ School of Public Health, North Sichuan Medical College, Nanchong, China; ^5^ Department of Clinical Medicine, North Sichuan Medical College, Nanchong, Sichuan, China; ^6^ Department of Information, Medical Support Center, Chinese People’s Liberation Army The General Hospital of Western Theater Command, Chengdu, Sichuan, China; ^7^ Institute of Basic Medicine, North Sichuan Medical College, Nanchong, Sichuan, China

**Keywords:** CAR T-cell therapy, BsAb, relapsed/refractory follicular lymphoma, efficacy, safety, cytokine release syndrome, neurotoxicity

## Abstract

**Background:**

Relapsed/refractory follicular lymphoma (R/R FL) remains a significant challenge in oncology, particularly for patients who have exhausted standard treatment options. Both chimeric antigen receptor (CAR) T-cell therapy and bispecific antibodies(BsAb) have emerged as promising therapeutic modalities in this setting, offering novel mechanisms of action and the potential for improved outcomes. However, comparative data on the efficacy and safety of these treatments remain limited. This study aims to evaluate the clinical outcomes and safety profiles of CAR T-cell therapy versus BsAb as third- or later-line treatments for R/R FL.

**Methods:**

A systematic review and meta-analysis were conducted to compare the efficacy and safety of CAR T-cell therapy and BsAb in patients with R/R FL. Studies were selected based on predefined inclusion criteria, and relevant data were extracted to assess overall response rates (ORR), complete remission (CR) rates, progression-free survival (PFS), and the incidence of adverse events, including cytokine release syndrome (CRS) and neurotoxicity. Statistical analyses were performed using random-effects models to account for variability across studies.

**Results:**

The analysis included 12 studies, with a total of 1,200 patients. CAR T-cell therapy demonstrated superior efficacy compared to BsAb, with a higher ORR (92% vs. 77%)[95% confidence interval (CI) 0.77-0.90] (p= 0.01)and CR rate (82% vs. 65%) [95% CI 0.65-0.80] (p< 0.001). The median PFS was significantly longer for CAR T-cell therapy (15 months) compared to BsAb (9 months). Adverse events were more common in the CAR T-cell group, particularly neurotoxicity (7%[95% CI 0.02-0.13]). However, the overall safety profile was manageable, with most adverse events being grade 1–2 in severity. BsAb were associated with a lower incidence of severe adverse events but showed less favorable efficacy outcomes.

**Conclusions:**

Our meta-analysis suggests that CAR T-cell therapy demonstrates a trend toward improved efficacy outcomes compared to bispecific antibodies (BsAb) in R/R FL, with higher response rates and longer PFS. However, this observed advantage must be interpreted cautiously due to potential confounders, including imbalances in baseline tumor burden, prior treatment lines, refractoriness to prior therapy, and variations in bridging therapy protocols across studies. Notably, CAR T-cell therapy was associated with a higher incidence of severe adverse events, particularly neurotoxicity. These findings indicate that while CAR T-cell therapy represents a promising therapeutic strategy, its comparative benefits require validation in studies with matched risk populations and standardized protocols. Future research should prioritize risk-adapted treatment selection and toxicity mitigation strategies for high-risk cohorts.

**Systematic review registration:**

https://www.crd.york.ac.uk/PROSPERO/view/CRD420251107275, Identifier CRD420251107275.

## 1 Introduction

### 1.1 Rationale

Follicular lymphoma (FL) is the second most common type of non-Hodgkin’s lymphoma (NHL) (1, 2), approximately 35% of all NHLs (3), and is an inert class of disease that is incurable with the application of chemoimmunotherapy (CIT). Despite diagnostic advances, FL is usually diagnosed at a late stage, with less than 10% of cases in stages I and II at diagnosis. Approximately 70% of patients have bone marrow involvement and less than 20% present with B symptoms (3).FL occurs in germinal centers and is characterized by at(14;18) translocation, leading to BCL-2 overexpression. According to the fifth WHO classification, classic FL is more common, while follicular large B-cell lymphoma and FL with uncommon features represent rare subtypes (4).

Current treatment strategies for FL include rituximab as monotherapy or watchful waiting for asymptomatic patients (5, 6), rituximab in association with chemoimmunotherapy (7–9), and rituximab maintenance therapy (10, 11). Maintenance immunotherapy based on lenalidomide in combination with rituximab (12) or otolizumab (ZO) (13) has shown longer progression-free survival (PFS). For relapsed or refractory (RR) disease, second-line (2L) therapy may include retreatment with similar regimens or alternative combinations (14).

Nonetheless, FL treatment remains challenging because it tends to recur or is refractory to standard therapy, despite being slow-growing and initially responsive. Overall survival (OS) after first-line treatment of FL can be prolonged up to 25 years; however, this survival rate decreases with each subsequent line of therapy. A median OS of 5.8 years has been reported for patients receiving third-line therapy, which further declines to 3.6 years for patients receiving fifth-line therapy (15). PFS with subsequent treatment decreases dramatically after the first relapse (16, 17). Patients with disease progression (PD) within 24 months of first-line treatment (POD24) have significantly shorter OS (18).

This decline underscores the need for improved therapeutic strategies. In the last 5 years, there has been a proliferation of targeted therapies for R/R FL, including novel antibody-based therapies such as magrolimab (which directly targets CD47 on macrophages rather than FL cells), tafasitamab (a CD19-targeted antibody), polatuzumab vedotin (an antibody-drug conjugate targeting CD79b) (19) and Obinutuzumab (a glycoengineered type II anti-CD20 monoclonal antibody) (20). In addition, small molecule inhibitors targeting apoptosis-regulating pathways such as PI3K kinase (Idelalisib, copanlisib, and duvelisib)and BTK have been shown to hold promise as new strategies for FL management. In this regard, the ROSEWOOD trial (21) demonstrated significant efficacy of zanubrutinib in combination with ZO in patients with R/R FL who had received ≥ 2 lines of therapy. This study demonstrated that the combination therapy was superior to anti-CD20 monoclonal antibody monotherapy, with an overall remission rate (ORR) of 69% for ZO versus 46% for O (p=0.001). A notable breakthrough in the area of high epigenetic mutation rates in FL was the FDA’s approval of tazemetostat for the treatment of R/R FL after two prior lines of therapy. Recent results from a phase II trial showed that EZH2 (tazemetostat) is a key epigenetic driver in the pathogenesis of FL.Tazemetostat was well tolerated and effective in R/R FL patients, with an ORR of 69% (95% CI 53-82; 31 of 45 patients), median duration of response was 10·9 months in the EZH2mut cohort (22).

Exciting advances in R/R FL research have focused on immune-based therapies such as bispecific antibody (BsAb) constructs (23) such as Mosunetuzumab, Epcoritamab, Odronextamab and chimeric antigen receptor T cells (CAR Ts) (24) such as Axicabtagene-ciloleucel, Tisagenlecleucel, Lisocabtagene-maraleucel. Both types use well-known B-cell lineage markers to direct autologous T cells towards lymphoma cells. Several agents within each category have demonstrated remarkable effectiveness and acceptable safety profiles, leading to their accelerated approval by the US Food and Drug Administration for treating R/R FL after at least two previous therapies (25–29).Significantly, phase 3 confirmatory trials are investigating these agents for use in either the first or second line, which could influence later therapy options. We examine the data for each category of agents and emphasize important factors for advising and ordering treatment for patients with R/R FL (30).

### 1.2 Objectives

These new approaches utilize different mechanisms to enhance the immune response to FL cells and offer further promising avenues for treatment. Although both CAR T therapy and BsAb have shown significant potential in the treatment of R/R FL, the lack of direct head-to-head studies comparing the efficacy, incidence of serious adverse events, and prognosis of patients between the two poses a challenge for clinical decision-making. Previous reports in the relevant literature (31–33) compared the respective characteristics of individual CAR T and individual BsAb products in terms of efficacy and safety. However, these three reports only compare a single product as a representative of the two therapeutic modalities, and there is a lack of pooled analyses and comprehensive comparisons of existing studies on the two therapeutic modalities. Therefore, we conducted a comparative meta-analysis of CAR T-cell therapy and BsAb therapy. By evaluating and summarizing their combined efficacy and adverse event profiles with different CAR T products and different BsAb products, we provide a clearer perspective on the therapeutic value of these two therapies in the third or backline treatment of R/R FL, and assist clinicians in making more appropriate and prognostically valuable choices for their patients.

## 2 Methods

### 2.1 Ethical statement

This meta-analysis was conducted using previously published data and did not involve any new research involving human participants or animals. All included studies had obtained ethical approval from their respective institutional review boards as reported in the original publications. As this analysis utilized publicly available data, additional ethical approval was not required. In addition, this study has been registered in the PROSPERO database (Registration Number: CRD420251107275).

### 2.2 Literature search

Literature evaluating the efficacy and safety of R/R FL CAR T-cell therapy or BsAb for the treatment of relapsed/refractory follicular lymphoma was collected from the Pubmed, Embase, and Web of science databases. Primary keywords included “follicular lymphoma,” “chimeric antigen receptor,” and “BsAb,” as well as derivatives of each keyword. The time limit for the search was from the creation of the database to November 30, 2024, and the search was conducted manually for each keyword. In addition, we manually searched conference abstracts for unpublished studies from each conference. All retrieved literature was screened for potentially eligible studies and there was no language restriction of included studies.

### 2.3 Eligibility criteria

Prospective interventional clinical trials that determined the therapeutic dose and assessed the efficacy of CAR T-cells or CD20×CD3 bispecific monoclonal antibodies for the treatment of R/R FL were included for meta-analysis. Studies involving one of the following were excluded: (1) preclinical studies, case reports, and literature inconsistent with the direction of the study; (2) endpoint events that were unclear or inconsistent with the present study; (3) primary evaluation of other subtypes of lymphoma; (4) duplicative, incomplete data, studies that were not completed or for which the original data were not found; (5) studies in children; (6) primary evaluation of the efficacy of other combinations of drugs used in conjunction with each other or of radiotherapy; (7) Evaluation of the efficacy of retreatment using therapies with the same mechanism of action; (8) Results produced by different follow-up times in the same study population; (9) Dual-targeted or other targeted CAR T therapies or BsAb.

### 2.4 Data extraction and risk of bias assessment

Two investigators independently extracted data from the included trials, and disagreements were resolved through discussion. The systematic review and meta-analysis referred to data extraction guidelines and used a pre-designed form to extract data, including Regimen, First Author, Year of Publication, Phase, Sample Size, Median age (range, yr), Mean number of prior treatment lines, Stage III/IV (%), Prior ASCT (%), Refractory to last prior treatment (%), Patients with prior CAR T treatment (for BsAb studies), Age ≥65 year, ≥3 Previous Lines of Treatment, FLIPI high (≥3) at study entry (%) and High tumor bulk (GELF criteria)*(%). Efficacy outcomes included complete remission rate (CR), overall response rate (ORR) and 1year PFS, and ≥Gread 3 adverse event outcomes including CRS, incidence of neurotoxicity (immune effector cell⁃related neurotoxicity syndrome, ICANS), and infection. Two investigators independently assessed the potential risk of assessment bias (MINORS) for these studies using methodological indices for non-randomized controlled studies. The global ideal score for non-randomized controlled studies was 16.

### 2.5 Data synthesis and analysis

The primary outcome was CR rate (CR rates represent the best response), secondary outcomes ORR rate, 1-year PFS rate and grade ≥3 adverse events including CRS, ICANS and Infection were analyzed according to treatment. The analysis was performed by first calculating the combined effect sizes of the primary and secondary outcomes, as well as the corresponding 95% CIs and P-values according to the random-effects model and fixed-effects model, respectively, and then selecting the appropriate model according to the calculated I^2^ values; if the I^2^ value was less than 50%, the fixed-effects model was selected; if the I^2^ value was greater than or equal to 50%, the random-effects model was selected. The model was a meta-analysis of single-arm proportions, weighted to the binomial distribution model for the calculation of the combined weights of the effect sizes. To assess primary and secondary outcomes according to treatment category, Q-test was used for subgroup analysis. After selecting an appropriate model, the effects of factors on CR rates were further analyzed, and meta-regression analyses were also performed to assess potential moderators affecting CR rates and to adjust for these variables. These moderators included Median age (range, yr), mean number of prior treatment lines, Stage III/IV (%), Prior ASCT (%), Refractory to last prior treatment (%), patients with prior CAR T treatment (for BsAb studies), Age ≥65 yr, ≥3 Previous Lines of Treatment and FLIPI high (≥3) at study entry (%). Variables with a P value of <0.1 in univariate meta-regression were included in multivariate meta-regression analysis. Metareg functions were performed using meta-regression, and mixed-effects models were used to assess the effect of factors on CR rates by estimating coefficients for fixed effects. Heterogeneity was assessed using τ2 and I^2^ statistics. Sensitivity analyses were performed in 3 ways: (1) based on the assessment of bias, specifically excluding studies with scores <12; (2) comparing the results of the random-effects model with those of the fixed-effects model; and (3) excluding 1 study at a time and analyzing its effect on the primary outcome estimate to assess whether any of the studies exerted a dominant effect. Two-sided statistical tests were used, and P<0.05 was considered a significant difference. Meta-correlation analysis and meta-regression modeling were performed using STATA 18.0 software.

## 3 Results

### 3.1 Literature search

**Figure 1:**
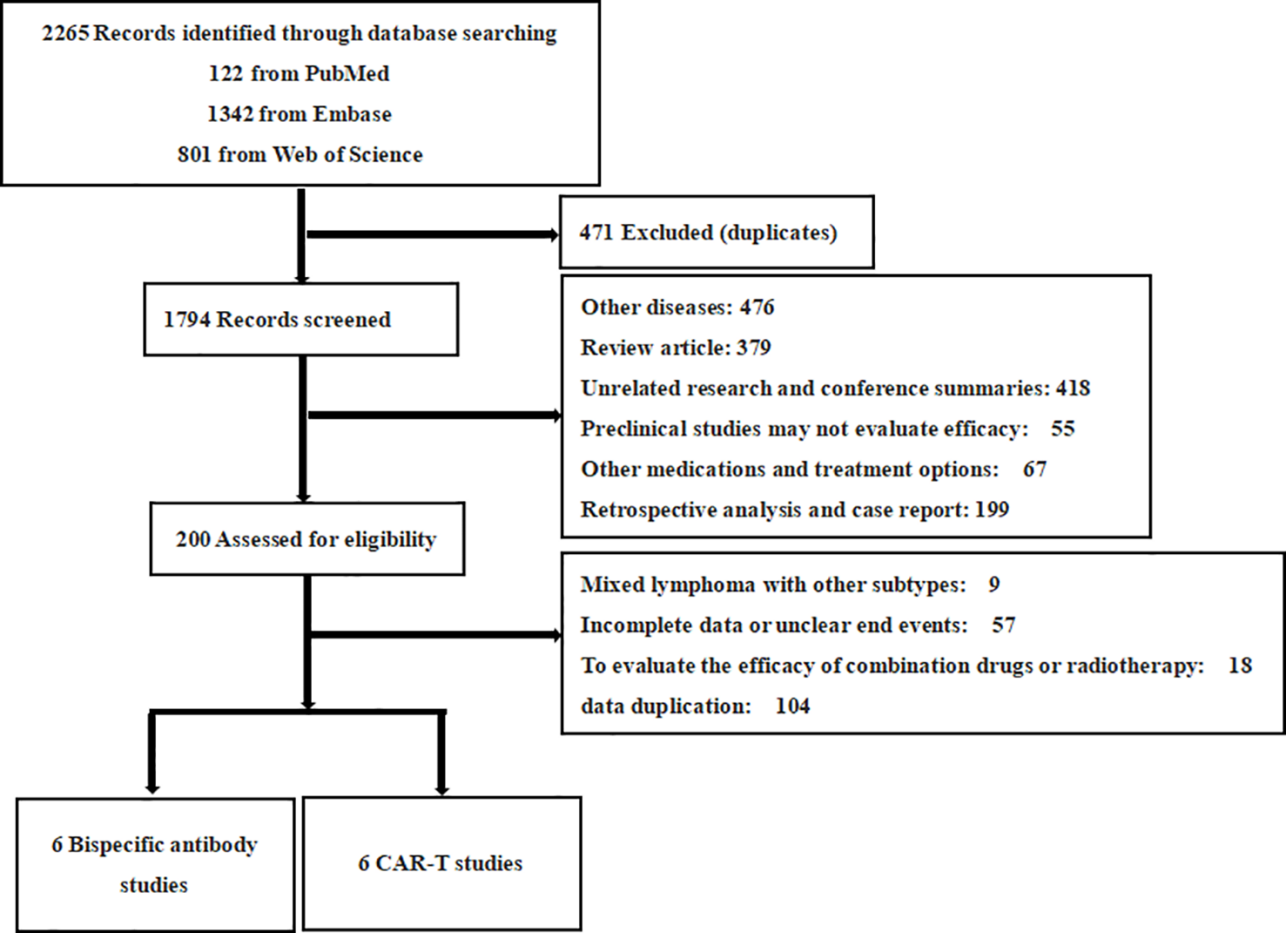
PRISMA flow diagram. This flow diagram depicts the systematic process of selecting studies for inclusion in the meta-analysis. A total of 2265 records were identified through database searching (PubMed: 122, Embase: 1342, Web of Science: 801). After excluding duplicates (471), 1794 records were screened. Following this, 200 records were assessed for eligibility, resulting in 12 eligible studies. These 12 studies consisted of 6 studies on BsAb and 6 studies on CAR T therapies. Excluded studies were primarily due to unrelated diseases, review articles, preclinical research, incomplete data, or other treatment methods.

The initial database search yielded 2,265 literature articles. Duplicates were excluded 471 studies and 200 studies that were still potentially eligible were excluded by reading titles and abstracts for detailed review. Finally, further exclusions were made based on the above inclusion ranking criteria, resulting in a final set of 12 studies, of which 6 involved CAR T therapy and 6 involved BsAb. All studies combined included 881 patients (**Figure 1**).

### 3.2 Study characteristics

The baseline characteristics of these 12 studies are detailed in **Tables 1** and **2**. with publication years from 2016 to 2024. 6 CAR T groups included 1 axi-cel (34), 1 Tisa-cel (25), 1 liso-cel (26), 1 CTL019 (35), 2 CD19 CAR T-cell groups alone (36, 37). 6 protocols of BsAb trials included 1 mosunetuzumab (27), 1 Epcoritamab (28), 2 odronextamab (38, 39), and 2 Glofitamab (40, 41).8 studies were phase 2 trials of all studies, except for two phase 1/2 studies (32, 33) and another two phase 1 studies (37, 38). Out of a total of 12 studies, 9 trials had a median of 3 prior treatments, of which 3 trials were in the CAR T-cell group (26, 34, 37), 6 trials were in the BsAb group (27, 28, 38–41), and the remaining 3 studies were in the CAR T-cell group, with two having a median of 4 at the treatment line (25, 36) and one having a median of 5 at the treatment line (35). In the BsAb trials, two studies (38, 41) excluded patients who had previously received CAR T-cell therapy, while the other four trials included patients with a history of CAR T, ranging from 3% to 29% of their populations. The risk of bias assessment is summarized in **Table 3**. A total score was also calculated to compare the quality of the studies, and sensitivity analyses were performed. Here, the studies we included were non-randomized controlled trials with a total score of 16, with a total score of 12–16 as excellent quality of A, a total score of 7–11 as good quality of B, and a total score of 0–6 as C, poor quality. The final scores of the studies we included ranged from 12 to 15, all of which were above excellent and of good quality. The main factors that lowered the overall score were item 6 (“whether the duration of follow-up was sufficient”), item 7 (“whether the follow-up dropout rate was less than 5%”), and item 8 (“whether the sample size was estimated “).

**Table 1:** Baseline characteristics of included studies.

	Trial Name	Authors (Trial identifier)	Phase	Regimen	No.	Median age (range, yr)	Stage III/IV (%)	Median No. Of previous therapy (range)	Prior ASCT (%)	Refractory to last prior treatment (%)	Chimeric antigen receptor T-cell therapy	Age ≥65 yr	≥3 Previous Lines of Treatment	FLIPI high (≥3) at study entry, n (%)	High tumour bulk (GELF criteria)*(%)
CAR T-cell															
1	ZUMA-5	Jacobson,CA, et al. (34) 2021 (NCT03105336)	2	axi-cel	124	60 (53–67)	0.85	3 (2–4)	0.24	0.68	NA	0.31	0.63	0.44	0.52
2	ELARA	Fowler, H, et al. (25) 2021(NCT03568461)	2	Tisa-cel	97	57(49–64)	0.856	4(2-13)	0.361	0.784	NA	0.247	0.278	0.598	0.639
3	TRANSCEND	Morschhauser, F, et al. (26) 2024 (NCT04245839)	2	liso-cel	107	62 (23–80)	0.88	3 (2–10)	0.31	0.64	NA	NA	0.5	0.57	0.53
4	NR	Chong, E, et al. (35) 2016(NCT02030834)	2	CTL019	14	59(43-72)	0.85	5(2-10)	0.21	1	NA	1	1	0.71	NA
5	NR	Hirayama, AV, et al. (36) 2019 (NCT01865617)	1/2	CD19	8	53 (49-57)	0.75	4 (2-7)	0.38	0.75	NA	0.13	1	0.5	0.25
6	NR	Shalev Fried, et al. (37) 2023(NCT02772198)	1b/2	CD19	26	62 (34 - 78)	0.85	3(2-6)	0.19	NA	NA	0.38	0.73	0.77	0.12
Bispecific antibody															
1	GO29781	Budde, LE, et al. (27) 2022 (NCT02500407)	2	mosunetuzumab	90	60 (53–67)	0.77	3 (2–4)	0.21	0.69	0.03	NA	0.62	0.44	0.34
2	EPCORE NHL-1	Linton, KM, et al. (28) 2024 (NCT03625037)	2	Epcoritamab	128	65 (55-72)	0.85	3 (2–4)	0.19	0.69	0.05	0.523	0.633	0.61	0.26
3	ELM-2	Kim, TM, et al. (38) 2024 (NCT03888105)	2	odronextamab	128	61 (22-84)	0.85	3 (2–13)	0.30	0.72	NA	NA	0.539	0.58	0.14
4	ELM-1	Bannerji, R, et al. (39) 2022(NCT02290951)	1	Odronextamab (REGN1979)	40	67 (57–73)	0.85	3 (2–5)	0.8	0.82	0.29	0.59	NA	NA	0.33
5	NP30179	Hutchings, Martin, et al. (40)2021 (NCT03075696)	1	glofitamab	44	64 (22-85)	0.778	3 (1-13)	0.24	0.906	0.018	NA	NA	NA	0.503
6	RG6026	Dickinson, M, et al. (41) 2021	2	glofitamab	75	64 (22-86)	0.77	3 (1-12)	0.24	0.90	NA	NA	NA	NA	NA

*High tumor bulk, as defined by any of GELF criteria, includes involvement of at least three nodal sites, each with a diameter of ≥3 cm; any nodal or extranodal tumor mass with a diameter of ≥7 cm.

**Table 2:** Efficacy and safety of included studies.

Authors (Trial identifier) R/R FL(3L+)	CR	Overall response rate	1 year PFS	CRS (Grade≥3)	Neurological events (Grade≥3)	Infection (Grade≥3)	OS (month 12/18)	median follow-up(month)
Jacobson, CA, et al. (34),2021 (NCT03105336)	0.79	0.94	0.68	0.06	0.15	0.18	0.93(12)	17.5
Fowler,NH, et al. (25),2021 (NCT03568461)	0.691	0.862	0.67	0	0.03	0.052	NA	16.59
Morschhauser, F, et al. (26),2024 (NCT04245839)	0.94	0.97	0.81	0.01	0.02	0.07	0.92(12)	18.9
Chong, E, et al. (35),2016 (NCT02030834)	0.64	0.79	0.77	0.14	0.07	NA	NA	11.4
Hirayama,AV, et al. (36),2019 (NCT01865617)	0.88	0.88	1	0	0	NA	NA	24
Shalev Fried, et al. (37),2023 (NCT02772198)	0.88	0.88	0.63	0.13	0.15	NA	1(12)	15.4
Budde, LE, et al. (27),2022 (NCT02500407)	0.6	0.569	0.577	0.02	0.01	0.14	0.89 (18)	18·3
Linton, KM, et al. (28),2024 (NCT03625037)	0.625	0.82	0.53	0.02	0	0.17	0.702 (18)	17·4
Kim, TM, et al. (38),2024 (NCT03888105)	0.73	0.80	0.662	0.017	0	0.28	0.701 (18)	20.1
Bannerji, R, et al. (39),2022 (NCT02290951)	0.72	0.91	0.60	0.06	0.03	0.12	NA	4.2
Hutchings, Martin et al. (40), 2021(NCT03075696)	0.477	0.705	0.486	0.023	0.012	0.175	NA	13.5
Dickinson, M, et al. (41),2021	0.693	0.813	0.79	0.05	0	NA	NA	8.6

**Table 3:** Quality evaluation (D1: Clear research purpose; D2: Continuity of inclusion of patients; D3: Prospective data collection; D4: Whether the end points are appropriate; D5: Objectivity of end points; D6: Whether the follow-up time is sufficient; D7: The loss of follow-up rate is less than 5%; D8: Whether the sample size was estimated; T: Total points).

Authors (Trial identifier)	D1	D2	D3	D4	D5	D6	D7	D8	T
Jacobson, CA, et al. (34),2021	2	2	2	2	2	1	2	2	15
Fowler, NH, et al. (25),2021	2	2	2	2	2	1	2	2	15
Morschhauser, F, et al. (26),2024	2	2	2	2	2	1	1	2	14
Chong, E, et al. (35),2016	2	2	2	2	2	1	1	0	12
Hirayama, AV, et al. (36),2019	2	2	2	2	2	2	1	0	12
Shalev Fried, et al. (37),2023	2	2	2	2	2	1	1	0	12
Budde, LE, et al. (27),2022	2	1	2	2	2	1	0	2	12
Linton, KM, et al. (28),2024	2	2	2	2	2	1	1	2	14
Kim, TM, et al. (38),2024	2	2	2	2	2	1	1	2	14
Bannerji, R, et al. (39),2022	2	2	2	2	2	1	1	2	14
Hutchings, Martin, et al. (40),2021	2	2	2	2	2	1	2	2	15
Dickinson, M, et al. (41),2021	2	2	2	2	2	1	2	1	14

### 3.3 Pooled efficacy outcomes

**Figure 2:**
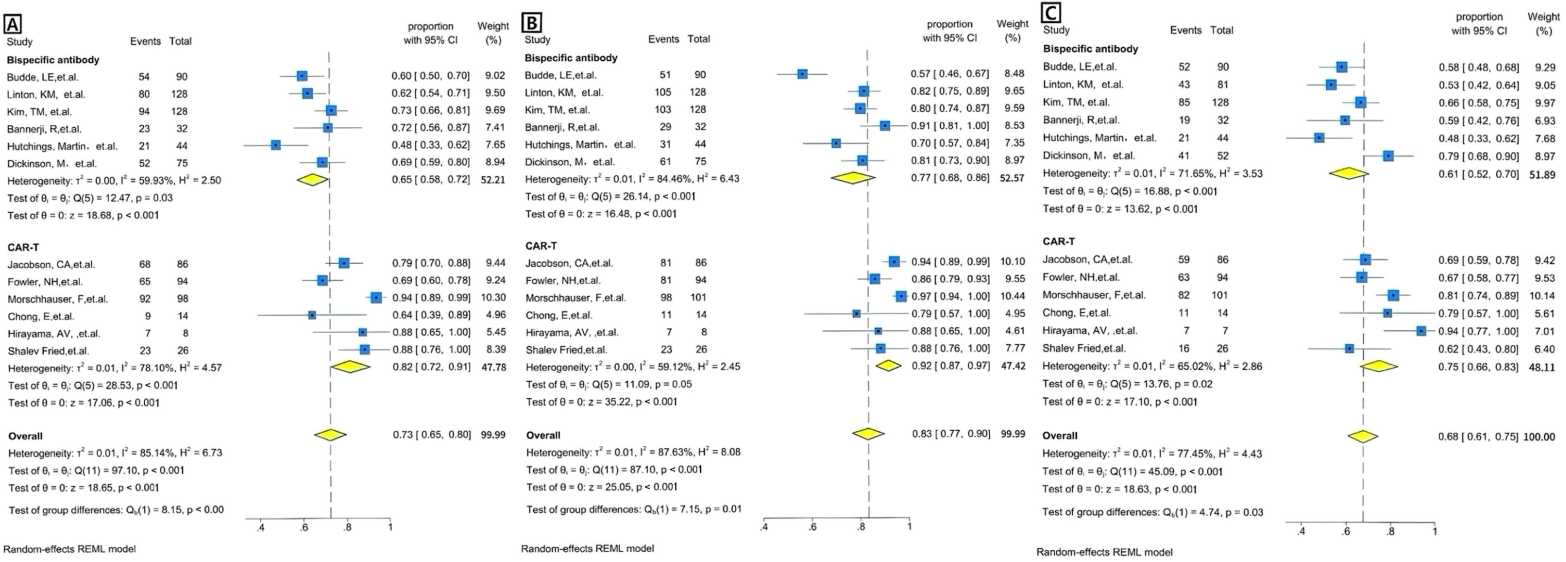
Forest plots of efficacy outcomes for BsAb vs. CAR T therapy. **(A)** Complete remission (CR): Forest plot showing the proportion of patients achieving complete remission (CR) across individual studies using BsAb in the upper and CAR T therapy in the lower. The weighted average proportion with 95% CI is indicated for each study. The pooled estimate is represented by the diamond. Statistical heterogeneity (I²=59.93%), significance (p=0.03) for BsAb, and statistical heterogeneity (I²=78.10%), significance (p<0.001) for CAR T therapy are reported. The overall pooled proportion of CR is displayed. The pooled heterogeneity for two Therapies (I²=85.14%) is also included, along with a significant p-value of <0.001. **(B)** Overall response rate (ORR): Forest plot showing the proportion of patients achieving overall response rate(ORR) across individual studies using BsAb in the upper and CAR T therapy in the lower. The weighted average proportion with 95% CI is indicated for each study. The pooled estimate is represented by the diamond. Statistical heterogeneity (I²=84.46%), significance (p<0.001) for BsAb, and statistical heterogeneity (I²=59.12%), no significance (p=0.05) for CAR T therapy are reported. The overall pooled proportion of ORR is displayed. The pooled heterogeneity for two Therapies (I²=87.63%) is also included, along with a significant p-value of =0.01. **(C)** 1 year PFS: Forest plot showing the proportion of patients achieving 1 year PFS across individual studies using BsAb in the upper and CAR T therapy in the lower. The weighted average proportion with 95% CI is indicated for each study. The pooled estimate is represented by the diamond. Statistical heterogeneity (I² =71.65%), significance (p<0.001) for BsAb, and statistical heterogeneity (I²=65.02%), significance (p=0.02) for CAR T therapy are reported. The overall pooled proportion of 1 year PFS is displayed. The pooled heterogeneity for two Therapies (I² =77.45%) is also included, along with a significant p-value of =0.03.

**Figure 3:**
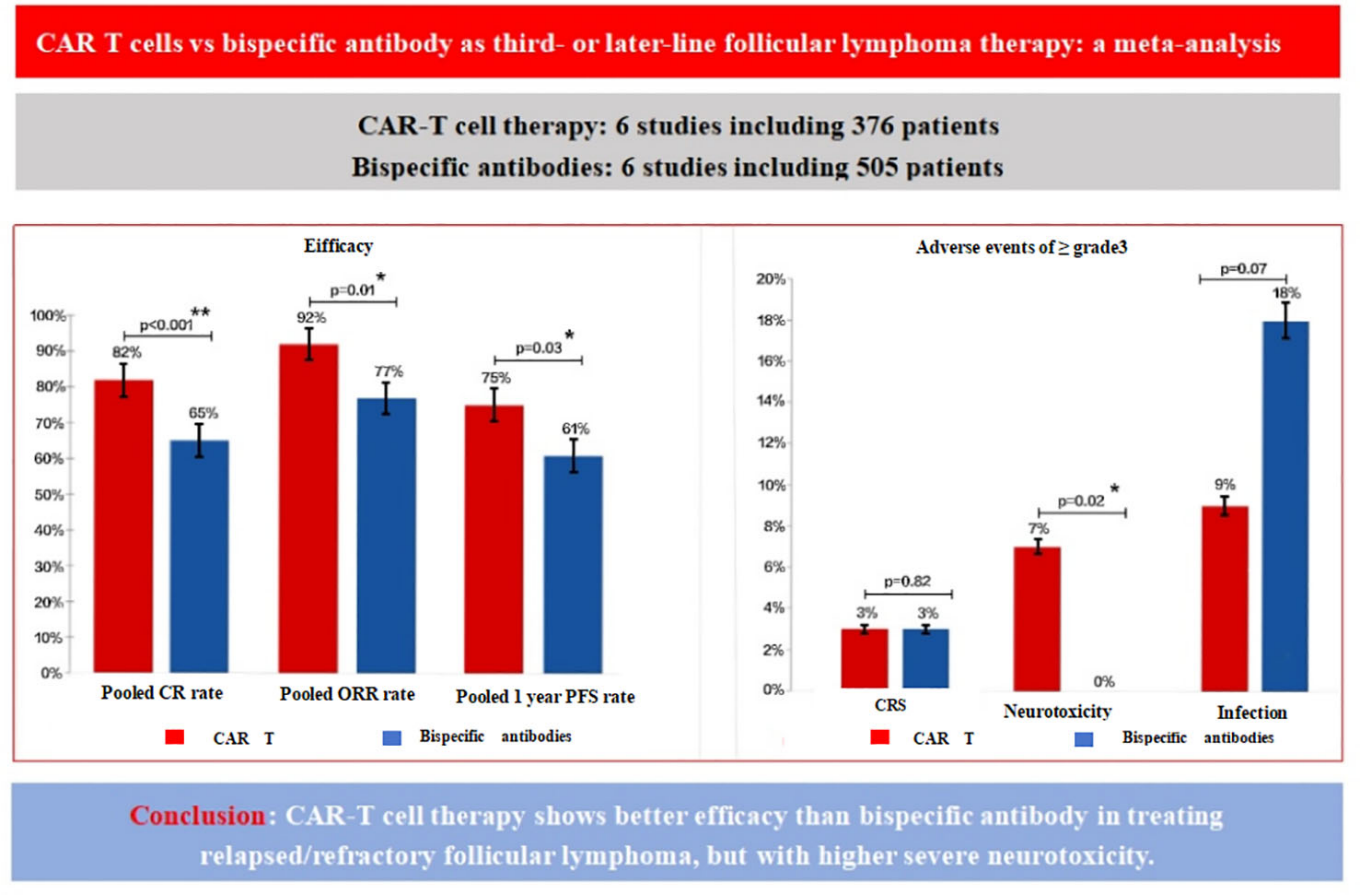
Comparison of efficacy and adverse events for CAR T-cells vs. BsAb. The figure compares the efficacy and adverse events between CAR T-cell therapy and BsAb therapy in the treatment of relapsed/refractory follicular lymphoma. The left panel presents efficacy outcomes, including the pooled complete remission (CR) rate, overall response rate (ORR), and one-year progression-free survival (PFS) rate. CAR T therapy shows superior efficacy with higher CR, ORR, and 1-year PFS rates [82%(p<0.001), 92%(p=0.05), and 75% (p=0.02), respectively], compared to BsAb, which have lower pooled rates [65%(p=0.03), 77% (p<0.001), and 61%(p<0.001)]. The right panel illustrates the incidence of grade ≥3 adverse events, showing that CAR T therapy is associated with a higher incidence of severe neurotoxicity [7% (p<0.001)], cytokine release syndrome (3% [p=0.04)], and infections (9%(p<0.001)) compared to BsAb [0 (p=0.43), 3%(p=0.18), and 18%(p=0.02), respectively]. These findings highlight the better efficacy of CAR T therapy, although with a higher risk of severe neurotoxic events.

We calculated both random effects model and fixed effects model results when combining effect sizes, but ultimately chose to use the random effects model because the calculations revealed heterogeneity between studies. The overall pooled proportion of CR was 0.73 [95% CI 0.65-0.80]. There was significant difference in CR rate between CAR T-cell therapy and the BsAb (p<0.001); 0.82 [95% CI 0.72-0.91] in the CAR T group and 0.65 [95% CI 0.58-0.72] in the BsAb group (**Figure 2A**). The overall pooled proportion of ORR was 0.83 [95% CI 0.77-0.90] and a notable difference in the ORR rate also existed between the two groups; 0.92 [95% CI. 0.87-0.97] for the CAR T-cell and 0.77 [95% CI, 0.68-0.86] for the BsAb (**Figure 2B**), with a significance level of p= 0.01. The overall pooled proportion of one-year PFS was 0.68 [95% CI 0.61-0.75] and a notable difference in the PFS rate also existed between the two groups; 0.75 [95% CI 0.66 -0.83] for the CAR T-cell and 0.61 [95% CI 0.52-0.70] for the BsAb (**Figure 2C**), with a significance level of p= 0.03, as shown in **Figures 2** and **3**.

### 3.4 Pooled safety outcomes

**Figure 4:**
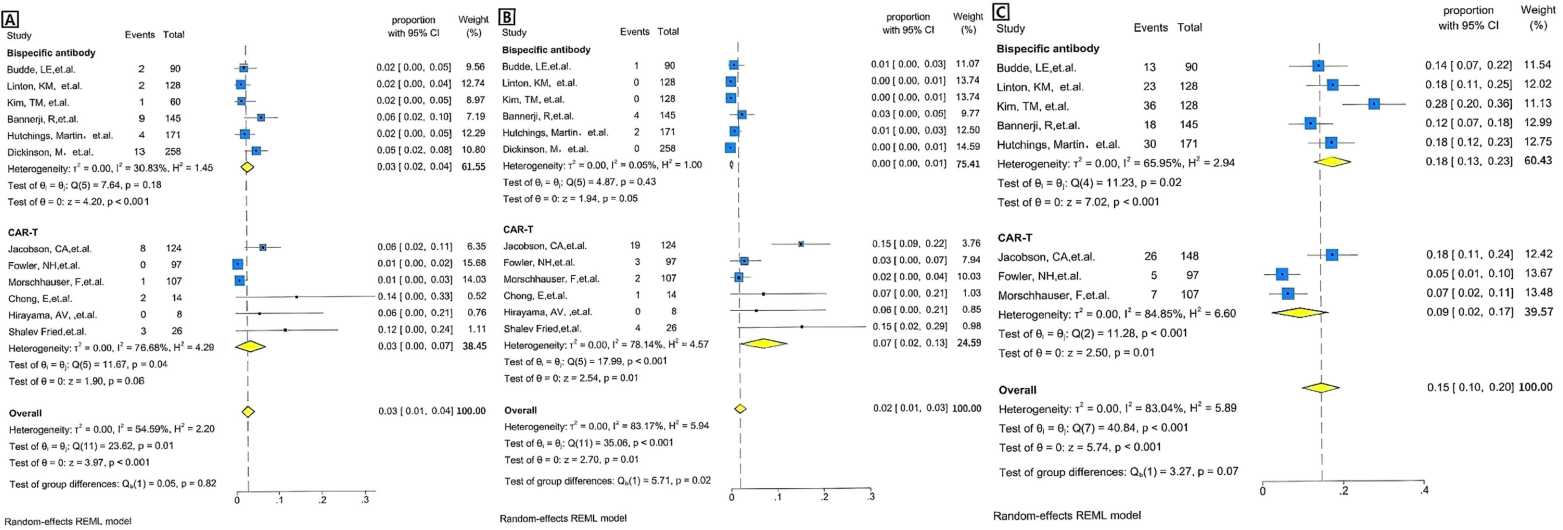
Forest plots of severe adverse events for BsAb vs. CAR T therapy, pooled grade ≥3 adverse events rate by the treatment category. **(A)** CRS: Forest plot showing the proportion of patients experiencing severe adverse events (e.g., CRS) across individual studies of BsAb in the upper and CAR T therapy in the lower. The proportion with 95% CI is displayed for each study, with the overall pooled estimate represented by the diamond. Statistical heterogeneity (I² =30.83%), no significance (p=0.18) for BsAb, and statistical heterogeneity (I²=76.68%), significance (p= 0.04) for CAR T therapy are reported. The overall pooled proportion of CRS is displayed. The pooled heterogeneity for two Therapies (I² =54.59%) is also included, along with no significant p-value of =0.82. **(B)** Neurotoxicity: Forest plot showing the proportion of patients experiencing severe adverse events (e.g., neurotoxicity) across individual studies of BsAb in the upper and CAR T therapy in the lower. The proportion with 95% CI is displayed for each study, with the overall pooled estimate represented by the diamond. Statistical heterogeneity (I² =0.05%), no significance (p=0.43) for BsAb, and statistical heterogeneity (I²=78.14%), significance (p<0.001) for CAR T therapy are reported. The overall pooled proportion of Neurotoxicity is displayed. The pooled heterogeneity for two Therapies (I² =83.17%) is also included, along with a significant p-value of =0.02. **(C)** Infection: Forest plot showing the proportion of patients experiencing severe adverse events (e.g., neurotoxicity) across individual studies of BsAb in the upper and CAR T therapy in the lower. The proportion with 95% CI is displayed for each study, with the overall pooled estimate represented by the diamond. Statistical heterogeneity (I² =65.95%), significance (p=0.02) for BsAb, and statistical heterogeneity (I²=84.85%), significance (p<0.001) for CAR T therapy are reported. The overall pooled proportion of Infection is displayed, no significance (p=0.07). The pooled heterogeneity for two Therapies (I² =83.04%) is also included.

The incidence of grade 3 or higher CRS was 0.03[95% CI 0.00-0.07] in the CAR T-cell group and 0.03[95% CI 0.02-0.04] in the BsAb group(**Figure 4A**). The overall pooled proportion of grade 3 or higher CRS was 0.03 [95% CI 0.01- 0.04] and no difference between the two groups with a level of p= 0.82. For neurologic events of grade 3 or higher, the rate was 0.07[95% CI 0.02-0.13] in the CAR T group, whereas the BsAb group presented an extremely low rate of 0.00[95% CI, 0.00-0.01] (**Figure 4B**). The overall pooled proportion of grade 3 or higher neurologic events was 0.02[95% CI 0.01- 0.03] and a notable difference between the two groups with a level of p= 0.02. As for infections of grade 3 or above, the CAR T-cell group reported an incidence of 0.09[95% CI 0.02-0.17] and the BsAb group showing a higher incidence at 0.18[95% CI 0.13-0.23] (**Figure 4C**). Nevertheless, the overall pooled proportion of grade 3 or higher infections was 0.15[95% CI 0.1- 0.2] and no difference between the two groups with a level of p= 0.07 (**Figure 3**).

### 3.5 Sensitivity analysis

To assess the robustness of the combined effect size, we pre-specified that a sensitivity analysis would be conducted by excluding studies with a relatively high risk of bias (defined as a MINORS score <12). However, as shown in **Table 3**, all 12 included studies had MINORS scores ranging from 12 to 15 (out of a maximum of 16), indicating high methodological quality. Therefore, no studies met the exclusion criteria, and a sensitivity analysis was not necessary.

### 3.6 Heterogeneity estimates

To assess whether the observed heterogeneity was influenced by variables such as patient characteristics or study factors and to adjust for these moderators, we developed a mixed-effects meta-regression model (**Table 4**). The univariate meta-regression indicated that CAR T-cell therapy (as opposed to BsAb) was a significant moderator associated with the key outcome CR rates. The 3 variables with p values below 0.1 in the univariate meta-regression included: CAR T-cell therapy (versus BsAb), stage III-IV (%), and last treatment refractory (%) (**Table 4**). The univariate meta-regression model incorporated the variable CAR T-cell therapy (odds ratio 0.1729 [95% CI 0.0572–0.2886]; p=0.003). However, when CAR T-cell therapy was included in the multivariate model, the effect did not reach statistical significance (odds ratio 0.1278 [95% CI -0.0091 to 0.2647]; p=0.067), suggesting that CAR T-cell therapy is not an independent predictor of CR rates. The decline in significance for all variables from univariate to multivariate analysis may be due to confounding interactions between the factors, which need further exploration.

**Table 4:** Meta‐regression analysis using study‐level characteristics in relation to CR.

Variables	Univariate analysis				Multivariate analysis			
	Coefficient	Standard error	95%CI	P	Coefficient	Standard error	95%CI	P
CAR-T cell therapy	0.1729	0.0590	0.0572 to 0.2886	0.003	0.1278	0.0698	-0.0091 to 0.2647	0.067
Stage III-IV(%)	1.4345	0.8296	-0.1915 to 3.0604	0.084	0.2936	0.8785	-1.4282 to 2.0155	0.738
Refractory to last prior treatment (%)	-0.6289	0.3518	-1.3183 to 0.0606	0.074	-0.4504	0.3436	-1.1239 to 0.2231	0.190
Median age(years)	-0.0101	0.0119	-0.0334 to 0.0133	0.398	NA	NA	NA	NA
Median No Of previous therapy	-0.0108	0.0753	-0.1583 to 0.1367	0.886	NA	NA	NA	NA
Prior ASCT(%)	0.0999	0.2626	-0.4147 to 0.6145	0.704	NA	NA	NA	NA
High tumour bulk (GELF criteria)*(%)	-0.0891	0.2793	-0.6365 to 0.4584	0.750	NA	NA	NA	NA

### 3.7 Publication bias

Publication bias was assessed through both visual inspection using a funnel plot and statistical tests, including Begg’s and Egger’s tests. The funnel plot suggested possible asymmetry, indicating potential publication bias, though visual interpretation is inherently subjective. Statistical tests did not confirm significant publication bias, with Begg’s test yielding a P-value of 1.000 and Egger’s test showing a P-value of 0.061 (**Supplementary Figure 1**). Furthermore, as a meta-analysis at the single-arm study level, we were unable to extensively investigate patient-level confounders and mediators.

## 4 Discussion

This meta-analysis highlights significant differences in efficacy and safety between CAR T-cell therapy and BsAb in R/R FL. The key outcome CR rate and secondary outcomes ORR and one-year PFS, all showed that CAR T-cells were superior to BsAb, which is the same conclusion as recently published literature (31, 32). In addition, regression analyses combined the variables of CAR T-cell therapy. The univariate analysis showed that CAR T-cell therapy was superior to bispecific antibodies (BsAb). However, after adjusting for potential confounders, including Stage III/IV (%), refractoriness to last prior treatment (%), median age (years), median number of previous therapies, prior ASCT (%), and high tumor bulk (GELF criteria) (%), the multivariate analysis revealed that CAR T-cell therapy did not remain a significant independent predictor of complete response (CR) (p=0.067). This suggests that other factors may influence the observed efficacy, and CAR T-cell therapy may not be an independent predictor when accounting for these confounders. This superior efficacy was accompanied by an increased incidence of high-grade neurologic events in the CAR T group, and there were no significant differences in the incidence of high-grade CRS and infections between the CAR T and BsAb groups. Thus, while CAR T-cell therapy represents a valuable therapeutic option for R/R FL, particularly in patients with high unmet needs or suboptimal responses to prior therapies, its dominant role must be contextualized within a framework that weighs efficacy against safety risks, logistical constraints, and equitable access. A personalized approach, considering both clinical characteristics and real-world implementation challenges, remains essential to optimizing patient outcomes in this setting.

In comparisons to mosunetuzumab (31) (n=90), axi-cel was associated with improved PFS (hazard ratio (HR)= 0.39, 95% CI[0.24-0.62]. Similarly, axi-cel led to higher ORR (odds ratio [OR]=3.87, 95% CI[1.53-9.76]) and complete response rate(CRR) (OR=2.80,95%CI[1.50-5.26]). Although axi-cel was associated with a higher rate of all-grade CRS (OR=5.54, 95% CI[2.97-10.35]) and neurological events(NEs) (OR=3.54,95% CI [1.28-9.83]), differences in grade 3 CRS and treatment-related adverse events(TRAEs) were not statistically significant. Findings from this study show improved efficacy and more durable response for the treatment of 3L+ R/R FL with axi-cel relative to mosunetuzumab, with increased odds of all-grade CRS and NE, but not G3+ CRS and TRAEs. Lisocel (32) was associated with higher ORR (OR=3.78, 95%CI[1.48–9.67]) and CR rate (OR=6.46, 95% CI 2.85–14.65), and improved DOR (hazard ratio [HR]=0.45, 95% CI 0.26–0.77) and PFS (HR=0.28, 95% CI 0.16–0.49) compared with mosunetuzumab. Results remained consistent across sensitivity analyses. Lisocel had a lower incidence of grade ≥ 3 CRS (OR=0.45, 95% CI 0.04–5.13), grade 3–4 serious infections. Tisagenlecleucel (33) produced statistically significant 11% higher ORR(91% vs 80%, P<0.05) and relative risk reduction in PFS events(HR=0.51, 95% CI [0.29, 0.87]; P<0.05), numerically better but not significant CR and OS (based on immature survival data), and similar safety outcomes vs mosunetuzumab in pts with r/r FL. Future analyses using IPD from both trials and real-world data are warranted.

CAR T-cell therapy modifies a patient’s T-cells to target the CD19 antigen on cancer cells (42). It has become an innovative therapy for the treatment of R/R lymphomas. Based on its promising results in pivotal trials, such as in tisa-cel, axi-cel and liso-cel, all of which have been approved by the FDA for third-line treatment of R/R FL. In particular, in the TRANSCEND trial (26), patients infused with liso-cel had a CR rate of 94% and an overall response rate (ORR) of 97%. In the NCT02030834 trial (35), patients infused with CTL019 had a CR rate of 64% and an ORR of 79%. The longest median follow-up was 24 months in the NCT01865617 trial (36), which had a CR and ORR of 88% and a 1-year PFS of 100%. These encouraging results suggest that CAR T-cells have the potential to change the therapeutic paradigm of R/R FL and instead position it as a second-line treatment option.

The emergence and authorization of CAR T-cell therapy has revealed the potential for T-cell-mediated treatment of B-cell malignancies, marking a significant advance in the treatment of patients with R/R FL. and a single CAR T infusion appears to be more convenient than prolonged BsAb administration. However, logistical and financial constraints, the time required for CAR T production, the specialized facilities required for CAR T-cell culture, and the difficulty of access in local hospitals and in many countries are significant barriers to CAR T therapy. In addition, CAR T is associated with a higher risk of developing any level of CRS and NEs (31) and often requires prolonged hospitalization. This limits its application to specialized centers with adequate resources. Infections are the leading cause of non-relapse mortality (43–45). Hematological toxicity is the most common adverse event after CAR T-cell therapy. Cytopenias can be profound and long-lasting (46, 47). Therefore, there is a need for off-the-shelf agents that provide durable remissions and are better tolerated. This need is particularly evident for patients whose disease progresses rapidly and requires immediate intervention, as well as for those who are elderly or have severe complications that do not tolerate intensive therapies.

In this context, another type of monoclonal antibodies known as BsAb, are monoclonal antibodies designed to target two different antigens (48). Available BsAbs in FL target CD20 on B cells and CD3 on T cells, triggering immune mediated tumor cell killing (49, 50). Currently, the mainstay BsAb being developed are those targeting CD20 on B cells and CD3 on T cells in the form of 1:1 or 2:1 CD20: CD3 antigen-binding fragments (51). BsAb are readily available, widely used in most centers, and associated with predictable low-grade CRS and very rare ICANS (52). This permits targeting of a wider patient population and a focus on outpatient treatment; however, training and close monitoring for CRS/ICANS and appropriate infection prevention measures are still required (53). The pharmacokinetics and safety of the subcutaneous route of administration, which may be associated with may be improved, thus further increasing the convenience of treatment. The efficacy of this therapy in R/R FL is remarkable, with CR rates ranging from 47.7% to 73.4%, ORR from 56.9% to 91%, and 1-year PFS from 48.6% to 79%. In contrast, CAR T patients had CR rates ranging from 64% to 94%, ORR ranging from 79% to 97%, and 1-year PFS ranging from 63% to 100%. The efficacy of the CAR T group is commendable. In the BsAb group, among patients who had received CAR T-cell therapy, the CR rates were 60% for mosunetuzumab (27), 62.5% for Epcoritamab (28), 72% for Odronextamab (39), and 47.7% for glofitamab (40). In our analysis, we found that the overall CR rate for the entire BsAb group was 65%, compared to 82% for the CAR T-cell group. Despite the fact that the BsAb group had received a median number of prior treatment lines of 3, which was less than the CAR T group, their CR rates were still significantly different compared to the CAR T group. This suggests that the overall tumor response was superior in the CAR T group. Compared with the mosunetuzumab group, the CART group had higher incidence of all-grade CRS and NE, but not G3+ CRS and TRAEs (31); lisocel had a lower incidence of grade ≥ 3 CRS, grade 3–4 serious infections (32); tisagenlecleucel had similar safety outcomes ^[337]^.

This meta-analysis showed that the odds of grade ≥3 neurologic events were higher in the CAR T group, whereas there was no significant difference in the odds of grade ≥3 CRS and infections between the CAR T and BsAb groups. From a clinical practice standpoint, the aforementioned findings establish a foundation for personalized treatment selection. For patients with pre-existing neurological conditions, advanced age, or limited tolerance to neurotoxicity, bispecific antibody therapy may represent a safer alternative. In the context of CAR T therapy, it is crucial to enhance dynamic monitoring of neurotoxicity, including regular assessments of consciousness, muscle strength, and electroencephalogram readings, and to commence the administration of IL-6 receptor antagonists, such as tocilizumab, at the earliest opportunity. It is important to note that this study did not reveal significant differences in CRS and infection-related adverse reactions. This finding implies that while CAR T therapy is associated with a heightened level of immune activation, bispecific antibody therapy may also provoke a certain degree of immune response. This finding underscores the critical necessity for stringent monitoring and prompt intervention in the management of CRS and infections within clinical environments, regardless of the therapeutic approach utilized, including CAR T cell therapy and bispecific antibody therapy. The implementation of systematic preemptive strategies is particularly vital for high-risk populations, such as the elderly and individuals with pre-existing medical conditions.

This meta-analysis assessed the quality of 12 included studies (6 CAR T and 6 bispecific antibody therapy studies), finding overall moderately high quality that supports the meta-analysis’s reliability. Across 8 dimensions (D1-D8) and total scores (T), the studies showed both common strengths and heterogeneities, reflecting current clinical research strengths and improvement areas. Notably, all studies scored perfectly (2/2) in core dimensions D1-D5, which cover clear objectives, standardized patient enrollment, prospective data collection, and scientific outcome evaluation. This consistency minimizes bias from design flaws and ensures result comparability. Heterogeneity emerged in dimensions D6-D8. For follow-up duration (D6), only Hirayama’s study scored 2/2, while 11 studies scored 1/2, indicating potential gaps in long-term outcome assessment. In loss to follow-up control (D7), Budde’s study scored 0/2 due to excess loss, while others scored 1-2/2, highlighting data integrity issues in some cases. Sample size estimation (D8) showed the largest discrepancy: 3 studies (Chong, Hirayama, Shalev Fried) scored 0/2 for non-standardized estimation, versus 9 studies scoring 2/2. Total scores ranged from 12-15, with Jacobson, Fowler, and Hutchings’ studies topping at 15 and Budde’s at 12. No quality differences were observed between CAR T and bispecific antibody studies, both showing “robust core dimensions but variable detail dimensions.” Despite limitations in follow-up and loss-to-follow-up management, perfect scores in D1-D5 confirm the included studies’ internal validity, providing a solid basis for subsequent efficacy and safety analyses.

Although the absence of a GRADE assessment limits our ability to fully evaluate evidence certainty, the current methodological rigor and study quality allow for confident, though cautious, interpretation of the results. Given the small sample sizes, non-randomized design, and the observed heterogeneity, we emphasize the need for further well-designed, larger studies to strengthen the overall conclusions and provide more precise estimates of the efficacy and safety of CAR T and bispecific antibody therapies.

This study has several inherent limitations. Firstly, despite multivariate adjustments for key potential confounders—including utilization of CAR T-cell therapy, proportion of stage III/IV disease, refractoriness to last prior treatment, median age, median number of prior therapies, prior ASCT rates, and high tumor bulk (defined by GELF criteria)—the comparative analysis of CAR T-cell monotherapy outcomes remains constrained by the limited number of eligible studies. This restricted sample size contributed to substantial heterogeneity within the monotherapy subgroup (quantified by Higgins I² > 50%), which may compromise the robustness of both subgroup-specific and pooled efficacy estimates. Future large-scale, prospective studies are warranted to validate these findings. Secondly, the P value of the Begg test was 1.000, and the P value of the Egger test was 0.061. Neither of them reached the significance level in the traditional statistical sense (P < 0.05). This result indicates that there is currently insufficient statistical evidence to support the existence of significant publication bias in this meta-analysis. However, it is worth noting that the P value of the Egger test is close to the critical value of 0.05, suggesting that if more research data are included in the future, the risk of bias may need to be re-evaluated. Thirdly, relatively few studies were included because of the lack of additional literature that could have been used to extract more useful data relevant to the analyses presented here. Fourthly, although significant heterogeneity was observed within groups based on different outcomes, no further analysis of heterogeneity, such as subgroup analysis within groups, was conducted. Fifth, given the exploratory nature of our study and limited reporting consistency across included trials, we did not formally apply the GRADE framework. Additionally, the lack of formal application of the GRADE framework is noted as a limitation of the study, which may impact the certainty of the evidence. This approach will be considered in future updates of the review. Despite these limitations, our meta-study has significant value. This is because we used a rigorous statistical methodology and a set of strict inclusion criteria that allowed us to confirm the relative efficacy of T-cell-mediated therapy in R/R FL. Finally, this study included relevant literature published before November 2024 for analysis, but did not include literature after November 2024 or ongoing clinical trials. This has a certain impact on the completeness of the study, but our research results still provide relatively reliable hints and guidance for the clinical decision-making of patients with R/R FL.

In conclusion, this pooled analysis showed that CAR T-cell therapy demonstrated a higher CR, ORR and one year PFS in third or subsequent lines of treatment compared to BsAb therapy, but was accompanied by an increased incidence of severe neurotoxicity (**Figure 3**).

## Data Availability

The original contributions presented in the study are included in the article/**Supplementary Material**. Further inquiries can be directed to the corresponding authors.
