# Supplementary material for: CAR T-cells vs. bispecific antibodies as third- or later-line treatment for relapsed/refractory follicular lymphoma: a literature review and meta-analysis
Source: Front Immunol. 2025 Sep 29;16:1611984. doi: 10.3389/fimmu.2025.1611984 (PMC12515661; doi:10.3389/fimmu.2025.1611984)
Supplement: Supplementary file 1 [file DataSheet1.pdf]

PRISMA 2020 Checklist

Section and Topic	Item #	Checklist item	Location where item is reported
**TITLE**			
Title	1	Identify the report as a systematic review.	Page1 lines 1-3
**AUTHER INFORMATION**			
Author information	2	Detailed description of author information.	Page1-4, Lines 4-111
**ABSTRACT**			
Abstract	3	See the PRISMA for Abstracts checklist.	Page5-6, lines 112-155
**INTRODUCTION**			
Rationale	4a	Describe the rationale for the review in the context of existing knowledge.	Page7-8，lines 156-215
Objectives	4b	Provide an explicit statement of the objective(s) or question(s) the review addresses.	Page 9，lines 216-232
**METHODS**			
Ethical Statement	5a	Provide an ethical statement if applicable.	Page10，Lines 234-240
Literature Search	5b	Present the full search strategies for all databases, registers and websites, including any filters and limits used.	Page10，Lines 241-250
Eligibility criteria	5c	Specify the inclusion and exclusion criteria for the review.	Page10-11，Line251-263
Data Extraction and Risk of Bias Assessment	5d	Describe the process for data extraction and risk of bias assessment.	Page11，Lines 264-279
Data synthesis and analysis	5e	Describe the methods for data synthesis and analysis.	Page11-12，Lines 280-310
**RESULTS**			
Literature Search	6a	Describe the results of the search and selection process, from the number of records identified in the search to the number of studies included in the review, ideally using a flow diagram.	Page13，Lines 312-318
Study Characteristics	6b	Cite each included study and present its characteristics.	Page13-14，Lines 319-341
Pooled Efficacy Outcomes	6c	Present meta-analysis results for efficacy outcomes (effect estimates, CIs)	Page14，Lines 342-356
Pooled safety Outcomes	6d	Present meta-analysis results for safety outcomes (adverse events, risks)	Page14，Lines 357-370
Sensitivity Analysis	6e	Present results of all sensitivity analyses conducted to assess the robustness of the synthesized results.	Page15，Lines 371-377

PRISMA 2020 Checklist

Section and Topic	Item #	Checklist item	Location where item is reported
Heterogeneity Estimates	6f	Present results of all investigations of possible causes of heterogeneity among study results.	Page15，Lines 378-392
Publication bias	6g	The current statistical results of all research publication biases.	Page15-16，Lines 393-401
**DISCUSSION**			
	7a	Summarize main findings and interpret in context of objectives.	Page16-20，Lines 402-544
	7b	Discuss limitations at study and review level.	Page20-21，Lines 545-577
	7c	Discuss implications for practice/research.	Page21-22，Lines 578-581
**OTHER INFORMATION**			
Authors' contributions	8	Describe each author's specific contributions to the work	Page23，Lines 582-586
Availability of data and materials	9	Declare availability of datasets, analytic code, and other materials	Page23，Lines 587-589
Funding	10	Describe sources of financial or non-financial support for the review.	Page23，Lines 590-595
Ethics approval and consent to participate	11	Describe ethical review and informed consent issues.	Page23，Lines 596-597
Consent for publication	12	State whether consent for publication was obtained	Page23，Lines598-599
Competing interests	13	Declare any competing interests of review authors.	Page23，Lines 600-601
Acknowledgments	14	Express gratitude to individuals/organisations that have provided assistance.	Page23，Lines 602-603
