# Supplementary figures and images for: CAR T-cells vs. bispecific antibodies as third- or later-line treatment for relapsed/refractory follicular lymphoma: a literature review and meta-analysis
Source: Front Immunol. 2025 Sep 29;16:1611984. doi: 10.3389/fimmu.2025.1611984 (PMC12515661; doi:10.3389/fimmu.2025.1611984)

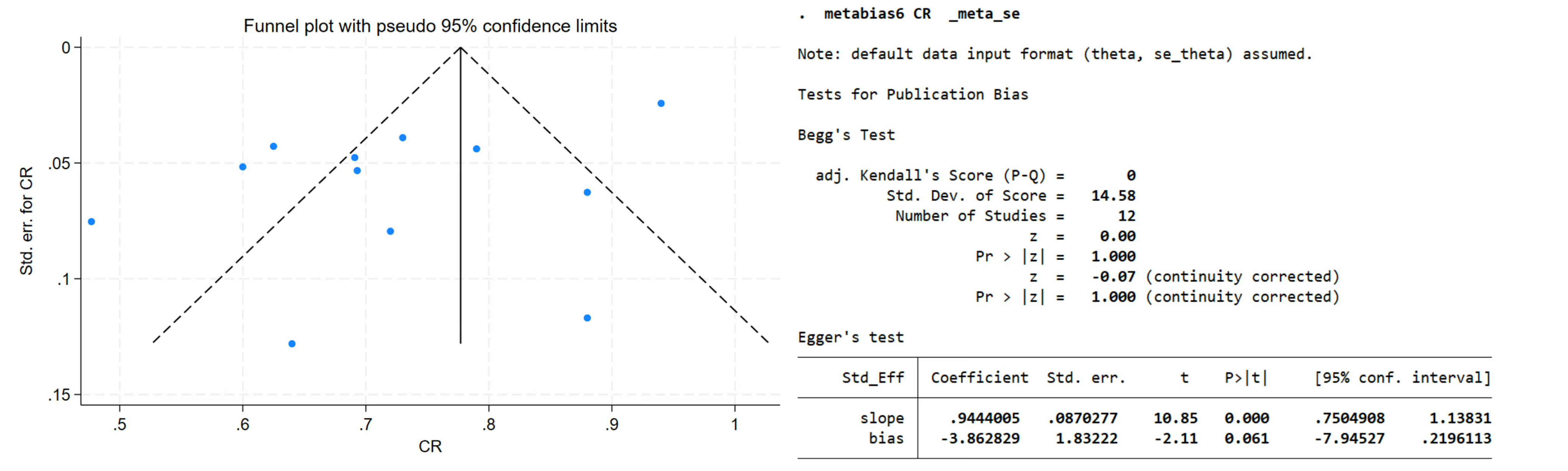

Supplement: Supplementary file 2 [file Image1.tif]
